# Light-dependent changes in the outer plexiform layer of the mouse retina

**DOI:** 10.3389/fopht.2023.1226224

**Published:** 2023-10-03

**Authors:** Tammie L. Haley, Ryan M. Hecht, Gaoying Ren, James R. Carroll, Sue A. Aicher, Robert M. Duvoisin, Catherine W. Morgans

**Affiliations:** Department of Chemical Physiology & Biochemistry, Oregon Health & Science University, Portland, OR, United States

**Keywords:** retina, photoreceptor, bipolar cell, ribbon synapse, light adaptation, immunofluorescence, electroretinogram (ERG), immunoblot (western blot)

## Abstract

The ability of the visual system to relay meaningful information over a wide range of lighting conditions is critical to functional vision, and relies on mechanisms of adaptation within the retina that adjust sensitivity and gain as ambient light changes. Photoreceptor synapses represent the first stage of image processing in the visual system, thus activity-driven changes at this site are a potentially powerful, yet under-studied means of adaptation. To gain insight into these mechanisms, the abundance and distribution of key synaptic proteins involved in photoreceptor to ON-bipolar cell transmission were compared between light-adapted mice and mice subjected to prolonged dark exposure (72 hours), by immunofluorescence confocal microscopy and immunoblotting. We also tested the effects on protein abundance and distribution of 0.5-4 hours of light exposure following prolonged darkness. Proteins examined included the synaptic ribbon protein, ribeye, and components of the ON-bipolar cell signal transduction pathway (mGluR6, TRPM1, RGS11, GPR179, Goα). The results indicate a reduction in immunoreactivity for ribeye, TRPM1, mGluR6, and RGS11 following prolonged dark exposure compared to the light-adapted state, but a rapid restoration of the light-adapted pattern upon light exposure. Electron microscopy revealed similar ultrastructure of light-adapted and dark-adapted photoreceptor terminals, with the exception of electron dense vesicles in dark-adapted but not light-adapted ON-bipolar cell dendrites. To assess synaptic transmission from photoreceptors to ON-bipolar cells, we recorded electroretinograms after different dark exposure times (2, 16, 24, 48, 72 hours) and measured the b-wave to a-wave ratios. Consistent with the reduction in synaptic proteins, the b/a ratios were smaller following prolonged dark exposure (48-72 hours) compared to 16 hours dark exposure (13-21%, depending on flash intensity). Overall, the results provide evidence of light/dark-dependent plasticity in photoreceptor synapses at the biochemical, morphological, and physiological levels.

## Introduction

The ability of the retina to adapt to different lighting conditions is essential to vision, and impairment of this ability reduces quality of life. Therefore, a better understanding of the cellular processes that regulate light sensitivity at all levels of the visual system is critical. Human vision operates over a vast 10^9^ log units of light intensity. To optimize vision over this entire range, the response properties of the retina change as a function of luminance at both the cellular and network levels. At the network level, the retina utilizes specialized circuits during the night (rods) and during the day (cones) (1). At the cellular level, molecular mechanisms of light adaptation are well understood in photoreceptor outer segments, where phototransduction takes place (2). In contrast, little is known about adaptation mechanisms at mammalian retinal synapses where photoreceptors contact their postsynaptic targets in the outer plexiform layer (OPL). Photoreceptor synapses represent the first stage of image processing in the visual system, and as such, adaptation mechanisms at this site are likely to have profound effects on vision.

**Figure 1:**
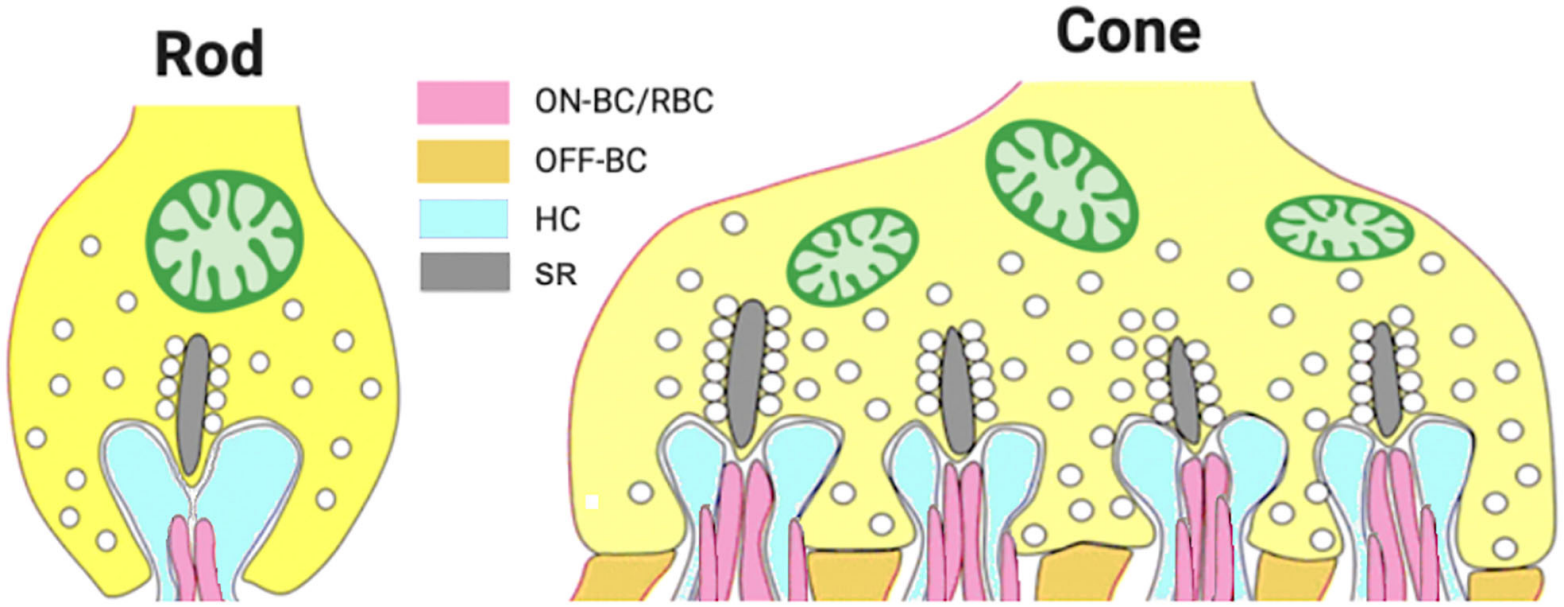
Diagram of photoreceptor synaptic terminals. Rod spherules have one active zone that contacts two horizontal cell dendrites and two rod bipolar cell dendrites. A single cone pedicle contains multiple active zones, each of which contacts two horizontal cell dendrites and dendrites of multiple subtypes of ON and OFF cone bipolar cells. RBC, rod bipolar cell; ON-BC, ON-bipolar cell; OFF-BC, OFF-bipolar cell; SR, synaptic ribbon.

Photoreceptors form biochemically and structurally specialized “ribbon synapses”, which support tonic glutamate release onto the dendrites of two classes of post-synaptic neurons: bipolar cells that transmit visual signals to the inner retina and horizontal cells that provide inhibitory feedback to cones and a feedforward signal to cone bipolar cells (**Figure 1**). Photoreceptors are hyperpolarized by light, depolarized by darkness, and modulate the rate of glutamate release in response to changes in light intensity. Rod synaptic terminals, called spherules, have a single active zone that contacts two rod bipolar cell (RBC) dendrites and two horizontal cell dendrites that protrude into an invagination of the spherule (**Figure 1**) (3). The active zone is defined by the synaptic ribbon to which synaptic vesicles are tethered and which runs perpendicular to the plasma membrane at the invagination. Cone terminals, called pedicles, are much larger and contain multiple active zones (~10 in mouse cones) (3), each with a synaptic ribbon and each contacting two horizontal cell dendrites and an array of bipolar cell dendrites (**Figure 1**). Cone pedicles make synaptic contacts with multiple cone bipolar cell types that fall into two broad classes, ON-bipolar cells and OFF-bipolar cells, that depolarize (ON) or hyperpolarize (OFF) to light according to the type of glutamate receptor they express. OFF-bipolar cells express ionotropic glutamate receptors, whereas ON-bipolar cells express a unique metabotropic receptor, mGluR6 (4). Similar to cone ON-bipolar cells, all RBCs are ON-type and utilize the mGluR6 signal transduction pathway. In all ON-bipolar cells, mGluR6 controls the gating of the TRPM1 cation channel via the heterotrimeric G protein, Go, and associated regulatory proteins, including RGS11 and GPR179 (reviewed in Martemyanov and Sampath, 2017; 5).

Electroretinogram recordings from mice and rats indicate that light- and dark-adaptation occur not only in photoreceptor outer segments, but also at photoreceptor synapses (6, 7). Additionally, electrophysiological studies using genetically-modified mice, in which key steps in retinal processing are inactivated, point to photoreceptor to ON-BC transmission as an important site of light-adaptation synaptic plasticity (8, 9). Photoreceptor synapses have a complex, yet stereotypical architecture that is conserved across species and intimately tied to the faithful encoding of luminance changes. Activity-driven changes in the biochemistry or geometry of photoreceptor synapses would directly affect synaptic transmission, and thus represent a potentially powerful, yet under-studied means of adaptation. To gain insight into mechanisms of adaptation in the OPL, we compared the morphology, as well as the abundance and distribution of key pre- and post-synaptic synaptic proteins under different states of light- and dark-adaptation.

## Materials and methods

To examine light-dependent changes in retinal proteins, C57BL6 mice were maintained on a 12 hr light-dark cycle, and retinas were removed after exposure to either 6-8 hours of indoor light (400 lux); 16, 24, 48, or 72 hours of darkness; or 72 hours of darkness followed by between 0.5-4 hours of light. To minimize circadian effects, light and dark exposures were timed such that all retinas were collected between 10 am and noon. Circadian effects cannot be ruled out entirely; however, as the period of most mice is less than 24 hours. Dark-adapted retinas were quickly removed (< 1 min) under dim red light (0.5 – 1.1 lux). Retinas were either immediately fixed for immunofluorescence and electron microscopy, or extracted with RIPA buffer for western blotting.

### Antibodies

Validation abbreviations are as follows: IB (immunoblot), IF (immunofluorescence), KO (knockout), HEK (transfected cells). Antibodies to RGS11, TRPM1 (sheep), and mGluR6 (mouse) were generous gifts of Ted Wensel (Baylor College of Medicine, Houston, TX), Kirill Martemyanov (Scripps Biomedical Research Institute, Jupiter, FL), and Melina Agosto (Dalhousie University, Halifax, Canada), respectively.

### Immunofluorescence

Light-adapted and dark-adapted retinas to be compared were processed in parallel, with cryosections mounted on the same slide and labeled with the same solutions. Retina sections were prepared and immunohistochemistry was performed as described previously (16) using primary antibodies listed in **Table 1**. Secondary antibodies were: anti-rabbit IgG, anti-mouse IgG, anti-sheep IgG, and anti-human IgG conjugated to either Alexa Fluor 594 or Alexa Fluor 488, (all used at 1:2000; Invitrogen, Grand Island, NY). Fluorescence images of retina sections were acquired with an Olympus FluoView FV1000 confocal microscope using a 60x/1.42 oil immersion objective. Brightness and contrast were enhanced using Adobe Photoshop (Adobe Systems Inc, San Jose, CA). Identical settings for image acquisition and enhancement were used for images that were to be compared to each other.

**Table 1:** Primary antibodies.

Antigen	Host	Validation	Use	Source (Catalog #) or Reference
PKCα	rabbit	IB, KO	IB, IF	Sigma-Aldrich (P4334)
PKCα	mouse	IB, KO	IF	Sigma-Aldrich, clone MC5
RGS11	rabbit	IB, KO, HEK	IB, IF	Chen et al., 2003 (10)
TRPM1	human	KO, HEK	IF	Xiong et al., 2013 (11)
TRPM1	sheep	IB, KO	IB	Cao et al., 2011 (12)
Recoverin	rabbit	IB	IF	Millipore (AB5585)
Ribeye/ctbp2	mouse	IB, HEK	IF	BD Biosciences (612044)
Ribeye, A domain	rabbit	IB, KO	IB	Synaptic Systems (192 103)
GPR179/cacna1S	mouse	IB, KO	IF	Abcam (ab2862); Hasan et al., 2016 (13)
Calbindin D	mouse	IB	IB, IF	Invitrogen (MA5-24135)
Goα	mouse	IB	IB, IF	Sigma-Aldrich (MAB3073)
mGluR6	sheep	IB, HEK, KO	IF	Morgans et al., 2006 (14)
mGluR6	mouse	IB, KO	IB	Agosto et al., 2021 (clone 312; (15)

### Western blotting

Retinal extracts were subjected to electrophoresis on precast 4% to 12% polyacrylamide gradient gels (Novex; Invitrogen, Carlsbad, CA). The separated proteins were electrophoretically transferred to PVDF membranes, which were probed with different antibodies, as previously described (14). Secondary antibodies conjugated to IR dyes were used at a dilution of 1:10,000, and visualized with an infrared imaging system (Odyssey; Li-Cor, Lincoln, NE). Band intensity was quantified with Licor Image Studio.

### Electroretinogram

Electroretinograms (ERGs) were recorded from WT C57BL6 mice as previously described (17). Mice were dark-adapted and then prepared for recording under dim red light. Mice were anesthetized with an intraperitoneal injection of ketamine and xylazine (100:10 mg/kg) and maintained with supplemental 30:3 mg/kg anesthesia injections approximately every 35 minutes. Body temperature was maintained at 36 — 37°C by placing the mouse on a circulating-water heating pad. Before ERG recording, the pupils were dilated with 2.5% phenylephrine and 1% tropicamide and the cornea was anesthetized with 1.0% proparacaine. A custom made cone placed over the snout allowed delivery of O2 which helped minimize breathing artifacts during recording. The ERG was recorded from a platinum needle electrode bent at 90°, placed in contact with the center of the cornea with a small amount of 2.5% methylcellulose gel. A platinum ring reference electrode was placed around the eye and a ground electrode was placed in the tail. The mouse and heating pad were then advanced into a Ganzfeld diffusing sphere and light stimuli were provided by custom made LED photoflash units. The flash intensity could be controlled by altering flash duration (between 30 μsec and 1 msec) and current through the LED. A 3.0 log unit neutral density filter was used to further extend the flash intensity range. Flash intensities were measured using a photometer (Model IL1700; International Light, Newburyport, MA) fitted with a scotopic filter in integrating mode that gave results as scotopic (sc) candela second per square meter (cd-s/m2). Scotopic and photopic ERGs were amplified at a gain of 5000, and band-pass filtered (0.1 to 1k Hz). Data were acquired with a data acquisition board (sampling rate: 10 kHz; National Instruments, Austin, TX). Traces were recorded with customized software (ERGlab, Dr Richard Weleber, Casey Eye Institute, Portland, OR).

### Electron microscopy

Whole eyes were dissected and perforated through the peripheral cornea before being incubated in a fixative of 2% paraformaldehyde and 2% glutaraldehyde in 0.1 M phosphate buffer (PB) for 5 minutes. The eyes were then halved along the corneal periphery and the retinas were extracted. The isolated retinas were fixed overnight at 4°C, washed in PB, and incubated in 1% osmium in PB for 30 minutes. After three PB washes, retinas were dehydrated in an ethanol series (30%, 50%, 70%, 95% for 5 minutes; twice in 100% for 10 minutes), followed by two propylene oxide exchanges (10 minutes each), and an overnight incubation in a 1:1 mixture of propylene oxide and Embed812 resin. The next day, retinas were incubated in 100% resin for 2.5 hours, embedded, and baked at 60°C for 24 hours. Ultrathin sections (silver, 60 nm) were collected onto copper grids and imaged using an FEI Tecnai T12 operating at 80Kv.

## Results

Electrophysiological recordings of light responses from mouse rod bipolar cells (both patch-clamp recordings from single cells in retinal slices and *in vivo* electroretinograms) are often performed on animals that have been dark adapted for 12-24 hours. Yet, most immunohistochemistry and anatomy studies are performed on light-adapted retina. This difference in light/dark adaptation between the physiological and anatomical preparations prompted us to investigate light- and dark-dependent immunohistochemical and morphological changes in the outer plexiform layer of the mouse retina.

### Prolonged dark exposure changes the distribution and labeling patterns of key retinal proteins

**Figure 2:**
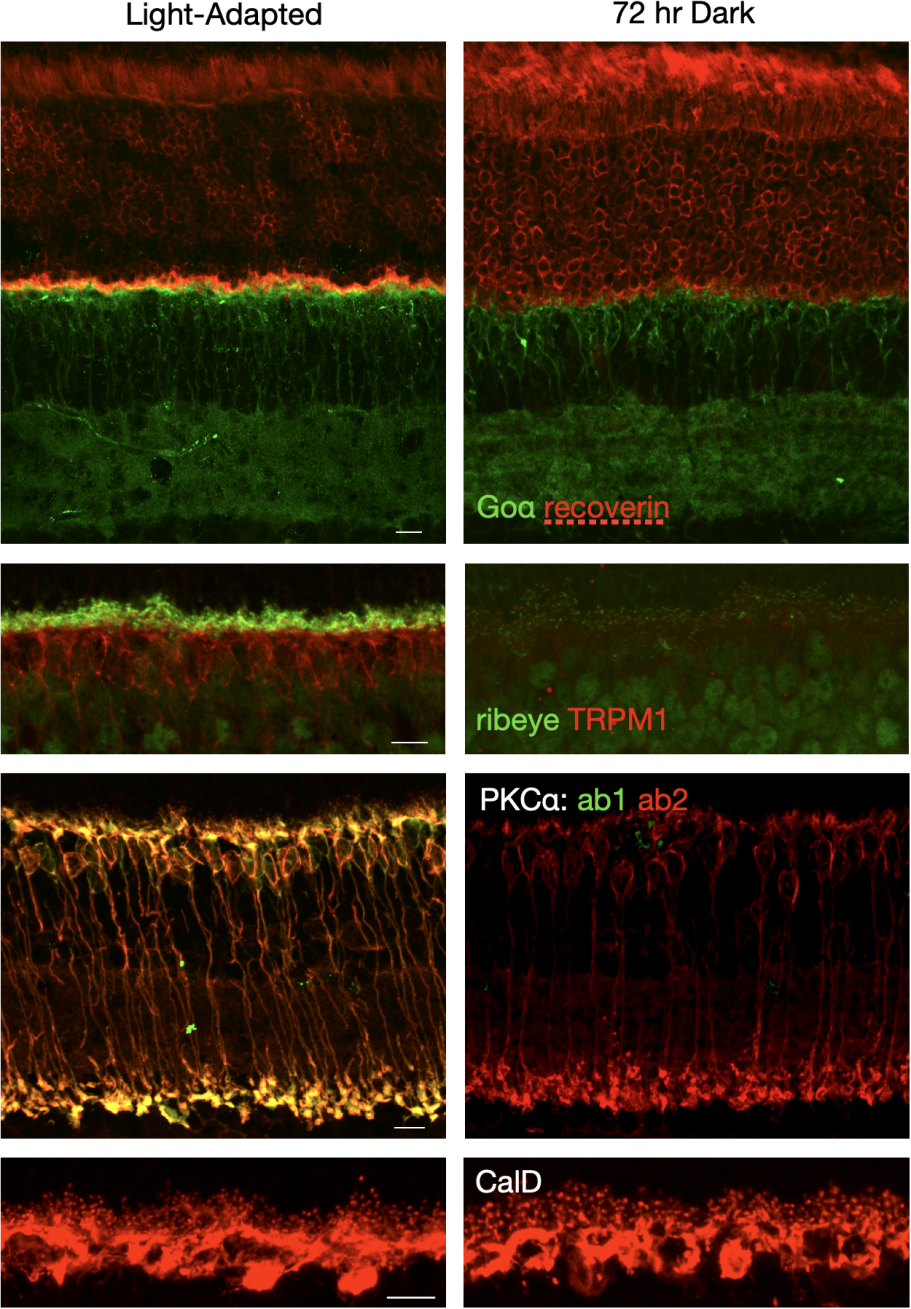
Immunofluorescent labeling of retinal proteins differs between the light-adapted state and prolonged dark adaptation. Retina sections from mice exposed to standard indoor light for 4-6 hours (light-adapted) or to 72 hours of darkness were double labeled with the following pairs of antibodies: PKCα - conformation-specific antibody (green) plus conformation-independent antibody (red), Goα (green) plus recoverin (red), ribeye (green) plus TRPM1 (red); and single labeled for calretinin (red). Confocal images were collected and processed using identical settings for each pair of light-adapted and dark-adapted retina sections. Scale bars represent 10 μm.

The distribution of retinal proteins was compared between light-adapted and dark-adapted states. Compared to the light-adapted retina, immunofluorescent labeling of synaptic proteins was largely unchanged following brief periods of dark adaptation (1-2 hours, not shown), but was dramatically reduced following prolonged dark exposure of 24-72 hours. **Figure 2** shows pairs of light-adapted (6-8 hr light) and dark-exposed (72 hr) retina sections labeled by immunofluorescence for PKCα (RBCs), calbindin D (horizontal cells), recoverin (photoreceptors), Goα (ON-BCs and inner retina), TRPM1 (ON-BC cell bodies and dendrites), and ribeye (photoreceptor and bipolar cell synaptic ribbons). The experiment was repeated three or more times for each antibody with similar results each time. Recoverin immunofluorescence revealed a change in the distribution of recoverin labeling from photoreceptor outer segments in the dark exposed state to photoreceptor synaptic terminals in the light-adapted state, similar to what has been previously described (18). Goa immunofluorescence appeared brighter over the ON-BC dendrites in the light-adapted retina compared to the dark-exposed retina. PKCα labelling was performed with two antibodies: a mouse monoclonal that is conformation-dependent plus a rabbit polyclonal that is conformation-independent. The monoclonal antibody binds to the hinge region of PKCα that is accessible when PKCα binds calcium in its active state. As we have demonstrated previously (19), both antibodies label RBCs in the light-adapted state, but only the conformation-independent antibody labels RBCs following prolonged dark exposure. Immunofluorescent labeling of horizontal cells with the calbindin D antibody was similar in intensity between the light-adapted and dark-exposed states, but the labeling pattern changed. Horizontal cell dendritic tips appear larger in the retina subjected to prolonged dark exposure, consistent with previous reports of light-dependent changes in horizontal cell morphology (20, 21). The most dramatic difference was observed for ribeye and TRPM1; labeling for both was substantially reduced in the retina subjected to prolonged darkness. Both proteins are involved in synaptic transmission in the outer plexiform layer between photoreceptors and ON-BCs, with ribeye, the main structural component of the synaptic ribbons, localized presynaptically, and TRPM1, the ON-BC transduction channel, localized post-synaptically. TRPM1 and ribeye immunofluorescence following 24 hours and 48 hours of dark exposure produced similar results as 72 hours dark exposure, but with slightly more ctbp2 labeling at 24 hours (data not shown).

### Light exposure following prolonged darkness rapidly restores the distribution and labeling intensity of ON-BC proteins to the light-adapted state

**Figure 3:**
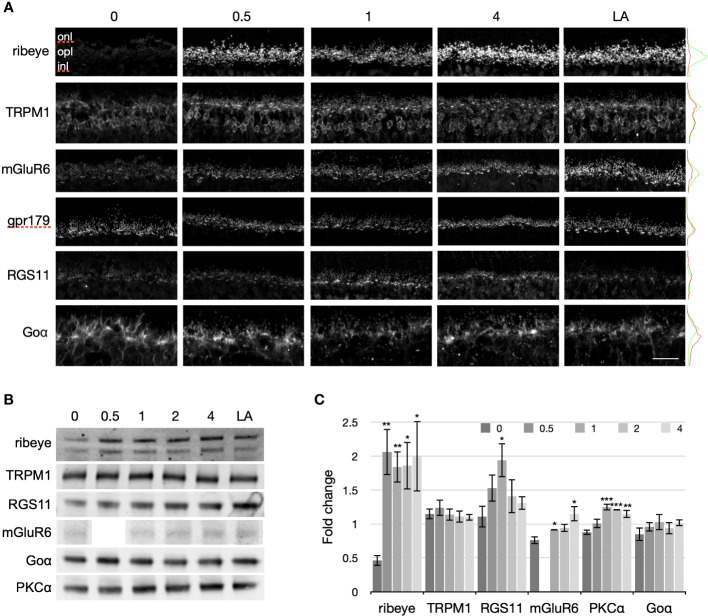
Light-dependent changes in proteins at photoreceptor to ON-BC synapses. **(A)** Immunofluorescent labeling of the different proteins was compared in retinas from mice following normal light adaptation (LA: 12 hr dark + 6-8 hr indoor light), or 72 hours of darkness followed by 0, 0.5, 1, or 4 hours of exposure to indoor light. The proteins examined included a presynaptic ribbon protein (ribeye) and postsynaptic components of the ON-BC signal transduction pathway (TRPM1, mGluR6, GPR179, RGS11, and Goα), as well as the RBC kinase, PKCα. To the right of each row of confocal images are fluorescence intensity profiles for the 0 hr (red) and light-adapted (LA, green) images. The scale bar represents 10 μm for the Goα images and 20 μm for all other images. Retina sections from 4 mice were labeled for each condition. For each antibody, labeling was similar across the quadruplicate sections and representative images are shown. **(B)** Immunoblots of ribeye, PKCα and ON-BC proteins (TRPM1, mGluR6, RGS11, and Goα) from mice that were either light-adapted or dark exposed for 72 hours followed by 0, 0.5, 1, 2, or 4 hours of exposure to indoor light. To compare changes in protein concentration between the different states, equal quantities of protein were loaded per lane. Each sample was prepared from four retinas from four mice (the other retina from each mouse was used for the immunofluorescence in **(A)**). **(C)** Immunoblot bands were quantified by densitometry and normalized to the light-adapted levels. Standard errors were derived from quadruplicate blots (except for ribeye, which used triplicate blots). Significant differences relative to the 72 hr dark-exposed state are indicated with asterisks above the error bars (*P ≤ 0.05, **P ≤ 0.01, ***P ≤ 0.001).

We next examined the transition from the prolonged dark-exposed state to the light-adapted state for ribeye, TRPM1, Goα, and PKCα, as well as additional proteins involved in the ON-BC light response (mGluR6, RGS11, and GPR179). Mice were either light-adapted (6-8 hours) or exposed to 72 hours of darkness followed by 0, 0.5, 1.0, 2.0, or 4.0 hours of light. To assess light-dependent changes in protein distribution and abundance, retinal cryosections were labeled by immunofluorescence for ribeye, TRPM1, mGluR6, GPR179, and RGS11 (**Figure 3A**). To measure light-dependent changes in protein expression, retinal extracts were immunoblotted for ribeye, TRPM1, RGS11, mGluR6, Goα, and PKCα, and immunoreactive bands quantified by densitometry (**Figures 3B, C**). **Figure 3B** shows examples of immunoblots for each protein and **Figure 3C** shows the quantification from quadruplicate blots (triplicate for ribeye) with protein levels normalized to the light-adapted mean.

Ribeye: As in **Figure 2**, ribeye immunofluorescence almost disappeared after 72 hours of dark exposure compared to the light-adapted state (**Figure 3A**, top row). Surprisingly, 30 min of light was sufficient to restore ribeye immunofluorescence to light-adapted levels. Immunoblotting revealed a decrease in ribeye expression of approximately 50% in the prolonged dark-exposed state compared to the light-adapted state. Following 72 hours in the dark, light exposure rapidly increases the intensity of the ribeye bands, so that only 30 min in the light increases ribeye immunoreactivity approximately four times that detected after prolonged darkness and 2 times that under normal light-adapted conditions. Ribeye immunoreactivity remained similarly elevated from 30 min to 4 hours of light exposure.

TRPM1: Overall, TRPM1 immunofluorescence was dimmer following prolonged dark exposure compared to any of the light-exposed timepoints (**Figure 3A**, second row). The most striking difference in TRPM1 labeling between the dark-exposed and light-adapted retina sections was the marked reduction of TRPM1 labeling in ON-BC dendritic tips in the 72 hr dark-exposed retina sections. This is especially obvious for RBCs, whose labeled dendritic tips dot the OPL in all of the light exposed sections, but are barely visible in the 72 hour dark exposed section. TRPM1 labeling associated with cone ON-BC dendrites appears as bright bars of labeling in the OPL of light exposed sections. Similar to the TRPM1 labeling of RBCs, TRPM1 immunofluorescence associated with cone ON-BC dendritic tips was much reduced following 72 hours in the dark compared to the light-adapted state. TRPM1 labeling of ON-BC cell bodies was also reduced following 72 hours of dark exposure, though the reduction in somatic labeling was variable between experiments (for example, compare TRPM1 labeling in **Figures 2**, **3**). As for ribeye labeling, 30 min of light exposure following 72 hours of darkness was sufficient to produce the light-adapted pattern of TRPM1 labeling, with strong immunofluorescence in ON-BC dendritic tips. Immunoblotting (**Figures 3B, C**) revealed that the amount of TRPM1 is unchanged by light or dark exposure, indicating that the light-dependent changes in immunofluorescence in **Figures 2**, **3A** are unlikely to reflect changes in protein concentration. Rather, the decrease in immunofluorescence following prolonged darkness may indicate a change in the protein that prevents antibody binding, such as a change in conformation, binding to another protein, or a post-translational modification such as phosphorylation.

mGluR6, GPR179, RGS11: Antibodies to all three ON-BC transduction proteins labeled RBC and cone ON-BC dendritic tips in the OPL in light-exposed conditions as well as following prolonged dark exposure (**Figure 3A**, 3rd - 5th rows). Labeling for mGluR6 appeared stronger with increasing light exposure. GPR179 immunofluorescence changed little between the dark- and light-exposed states. For RGS11, labeling of light-adapted sections and 72 hour dark-exposed sections was similar, but 0.5 to 4 hrs in the light following 72 hours of dark exposure increased the intensity of RGS11 labeling, with the strongest labeling occurring after an hour of light exposure. The changes in immunofluorescence labeling of retina sections correlated with changes in the retinal concentration of each protein as indicated by the band intensities in the immunoblots (**Figure 3B**).

### Electron-dense vesicles are present in dark-adapted ON-BC dendrites

**Figure 4:**
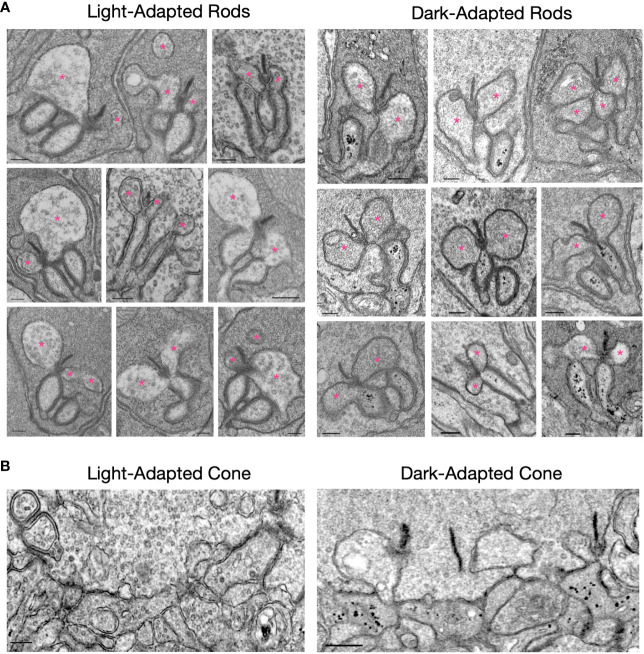
EM micrographs of light-adapted and dark-adapted photoreceptor active zones. **(A)** Rod spherules: Electron dense vesicles are present in rod bipolar cell dendrites in the dark-adapted state, but are rarely seen in the light-adapted state. Horizontal cell dendrites are indicated with pink asterisks. **(B)** Cone pedicles: electron dense vesicles are present in some post-synaptic processes in the dark-adapted state, but not in the light-adapted state. The scale bar in each image represents 200 nm.

The ultrastructure of rod and cone synapses from mice that were either light-adapted (6-8 hours) or dark-adapted (16-24 hours) was compared by electron microscopy. EM micrographs of rod spherules (**Figure 4A**) appear similar in the light- and dark-adapted conditions with respect to synaptic ribbons, synaptic vesicles, and the arrangement of RBC and HC dendritic tips within the spherules. A notable exception is the presence of numerous electron-dense vesicles in the dark-adapted RBC dendrites, which were rarely seen in the light-adapted retinas. Electron-dense vesicles were also present in post-synaptic processes at cone pedicles in dark-adapted but not light-adapted retinas (**Figure 4B**). The identity of these vesicles is unknown, but it is tempting to speculate that they may contain components of the mGluR6-TRPM1 signaling pathway that are being removed from the plasma membrane following extended dark exposure. This is supported by our observation that TRPM1 disappears from ON-bipolar cell dendritic tips following prolonged darkness (**Figure 3A**) despite no change in total TRPM1 levels (**Figures 3B, C**). Photoreceptor synaptic ribbons were prominent in both light-adapted and dark-adapted retina sections, suggesting that the dramatic decrease in ribeye immunolabeling in the dark-adapted retina (**Figure 3A**) is not due to disappearance of the ribbons.

### Prolonged dark exposure decreases ERG amplitudes

**Figure 5:**
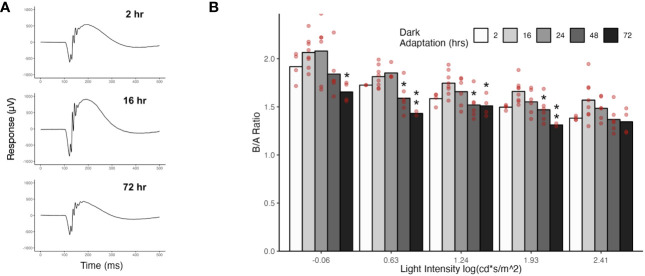
ERGs from mice following different dark exposure times. **(A)** Representative ERG traces from mice that were dark exposed for 2, 16, or 72 hours. Flash strength = -0.06 log(cd*s/m2^). **(B)** Scotopic ERGs were recorded from mice that had been dark exposed for 2, 16, 24, 48, or 72 hours. The mean b-wave to a-wave ratios are plotted for light intensities that gave a measurable a-wave (-0.06 to 2.41 log(cd*s/m^2)). Values for individual eyes are indicated with red circles. Significant differences relative to the 16 hr dark-adapted state (our standard dark-adaptation time) are indicated with asterisks (*P ≤ 0.05, **P ≤ 0.01).

We used electroretinogram recordings to assess the effect of dark-adaptation time on photoreceptor to ON-bipolar cell transmission. The ERG provides a physiological measurement of retinal function in response to a light flash. The initial component of the ERG waveform is a downward deflection termed the a-wave that is generated by photoreceptor hyperpolarization. The subsequent upward deflection is termed the b-wave and reflects the depolarization of ON-bipolar cells (22). Thus, the ERG waveform distinguishes between adaptation in phototransduction (which would affect both the a-wave and b-wave) from adaptation at the photoreceptor synapse (which would affect the b-wave without affecting the a-wave). For scotopic ERGs, mice are dark adapted and responses recorded to flashes against a dark background. The responses are driven by rods, with cones contributing at brighter flash intensities. Because the bipolar cell response is dependent on upstream photoreceptor activity, we normalized the b-wave amplitude to the a-wave amplitude for each light intensity by calculating the b-wave to a-wave ratio. We examined the effect of dark adaptation time on b/a-wave ratios by recording ERGs after 2, 16, 24, 48, or 72 hours of dark exposure. Representative ERG traces are shown in **Figure 5A** for 2, 16, and 72 hours of dark exposure. Dark adaptation of 16 and 24 hours produced the largest b/a waves, and this dark adaptation range (16-24 hours) corresponds to our standard scotopic ERG protocol. Prolonging dark exposure to 48 and 72 hours led to smaller b/a-wave ratios, with the smallest b/a ratios occurring following 72 hours of dark exposure, though the difference relative to 16 hours was not significant at the brightest intensity. Dark adaptation of 2 hours also resulted in smaller b/a-wave ratios relative to 16 hours, but the difference was not significant at any flash intensity.

## Discussion

We found marked changes in the expression and distribution of ON-bipolar cell proteins and proteins involved in photoreceptor to ON-bipolar cell transmission in animals subjected to prolonged dark exposure compared to light-adapted animals (**Figures 2**, **3**). Prolonged dark exposure led to a decrease in immunofluorescence for several key signal transduction proteins in ON-BC dendrites (TRPM1, RGS11, mGluR6) due to either an apparent reduction in protein concentration (RGS11 and mGluR6) or redistribution (TRPM1). Exposure to indoor light for 4 hours or less following the 72 hours in darkness largely restored the light-adapted pattern of immunolabeling (**Figure 3A**). This was particularly striking for ribeye and TRPM1 for which a light-adapted pattern of immunofluorescence required only 30 min of light exposure. Considering the decrease in signal transduction protein abundance in the ON-bipolar cell dendritic tips, mice exposed to 72 hours of darkness generated surprisingly robust ERG responses (**Figure 5**), with amplitudes similar to mice dark-adapted for only 2 hours and with b/a-wave ratios that were 79 - 87% (depending on flash strength) of those from mice dark-adapted for 16 hours. Our ERG findings contrast with a previous study on rats showing that the a-wave was little changed by 24 hours of dark exposure, but that the b-wave was substantially reduced in amplitude (6). The same study showed that if the prolonged dark exposure was interrupted by 30 min of light, the b-wave was quickly restored to its normal dark-adapted amplitude. This is consistent with our observations of reduced immunofluorescence of synaptic proteins in the outer plexiform layer following prolonged dark exposure and their rapid recovery upon exposure to light. In the present study, the ERG series is recorded over a range of light intensities involving multiple flashes, so it is possible that rapid light-dependent changes in synaptic proteins could take place during the ERG procedure affecting the amplitudes of later traces.

Intriguingly, TRPM1 showed almost no change between light- and dark-adapted samples by western blotting (**Figures 3B, C**), even though TRPM1 immunofluorescence was dimmer in the cell bodies and nearly absent from the ON-BC dendritic tips in the dark-exposed retina sections (**Figures 2**, **3A**). This suggests that there may be a pool of TRPM1 that is inaccessible to the antibody in the dark-exposed retina sections. It is tempting to speculate that the electron dense vesicles observed in dark-adapted rod bipolar cell dendrites in the electron micrographs may be transport vesicles shuttling TRPM1 away from the plasma membrane in the dendritic tips to an intracellular store in the cell body. Using high resolution confocal and STORM imaging, Agosto et al. (2018) (23) demonstrated that TRPM1 in ON-bipolar cell bodies is intracellular, residing in ER membranes that extend into the dendrites, but not to the dendritic tips (23). It is conceivable that plasma membrane insertion of TRPM1 is dynamically controlled as a means of adjusting synaptic gain with changes in background luminance.

Prolonged dark exposure led to a small decrease in PKCα, a kinase highly expressed in rod bipolar cells, to approximately 90% of light-adapted levels (**Figures 2**, **3C**). While the concentration of PKCα may change relatively little, we have previously demonstrated that PKCα activity in rod bipolar cells is strongly light-dependent. Using PKCα wild type and knockout mice and an antibody to phosphorylated PKC motifs, we showed that RBC dendritic tips are the major site of light-dependent PKCα phosphorylation (19). Light-dependent phosphorylation was also observed at cone synapses in both wild type and PKCα knockout retinas, suggesting that cone bipolar cells utilize a different member of the PKC family (19). In the present study, PKCα double labeling with conformation-specific and conformation-independent antibodies (**Figure 2**) confirms that PKCα is active in light-adapted retina and inactive following prolonged dark exposure, consistent with our previous findings. Thus, light-dependent kinase activity in ON-bipolar cell dendrites may drive light-dependent changes in activity or localization of other signal transduction proteins.

Immunofluorescent labeling for calbindin D revealed a possible difference in horizontal cell morphology in retinas from light-adapted mice compared to those from 72 hour dark-exposed mice, with the tips of the dark-exposed horizontal cells appearing larger than in the light-adapted state (**Figure 2**). Enlarged HC processes in response to dark exposure have been observed at the ultrastructural level (20, 21). Specifically, electron micrographs of rod spherules in the mouse retina found a 4-fold increase in the volume of HC endings within 30 min of dark exposure accompanied by a corresponding increase in the area of the rod plasma membrane lining the invagination (21), suggesting a tight coupling between the membrane dynamics of these compartments. EM studies of fish and amphibian retinas also demonstrate significant light-dependent changes in HC dendrites (24).

Presynaptically, dramatic changes in ribeye labeling were observed, with immunofluorescence being nearly undetectable after 72 hours of dark exposure compared to light-adapted conditions, but rapidly exceeding light-adapted levels after only 30 min of light exposure. These light-dependent changes in ribeye immunofluorescence are unlikely to represent actual changes in synaptic ribbon size; however, as EM micrographs of rod spherules (**Figure 4A**) reveal similar synaptic ribbons in light-adapted and dark-adapted conditions. Furthermore, 30 min is unlikely to be sufficient time for transcription, translation, protein trafficking and ribbon assembly to occur. An alternative explanation is that the rapid changes in ribeye immunoreactivity (**Figure 3**) may be due to light-dependent post-translational modification of ribeye that affects binding of the antibody, such as phosphorylation/dephosphorylation. Prior studies on the effects of light- and dark-adaptation on mouse photoreceptor synaptic ribbons report contradictory results. For example, Dembla and colleagues (2020) find that immunofluorescence labeling of ribeye is more intense in dark-adapted versus light-adapted conditions, and conclude that ribbons are larger in the dark-adapted state (25); but other studies found no difference between light- and dark adapted ribbons (20, 26). It is possible that the differences in dark exposure times and antibodies used may account for the seemingly conflicting results between studies.

Because photoreceptors depolarize to decreases in light intensity, they are often assumed to undergo maximal rates of synaptic vesicle cycling in complete darkness; however, this is an over simplification. While it may be true that they achieve maximum rates of glutamate release during brief periods of darkness, during prolonged darkness, rather than maintaining a maximum rate of transmission, synaptic activity is instead suppressed, possibly driven by the need to conserve energy. This is likely to be accompanied by down regulation of post-synaptic signaling pathways. This pre- and post-synaptic suppression of activity could occur via a variety of mechanisms that can be rapidly reversed upon light stimulation, such as removal of key proteins from the dendritic tips/active zone, phosphorylation/dephosphorylation, or interactions with regulatory proteins. In future studies, it will be interesting to correlate the effects of short-term dark- and light-adaptation on synaptic morphology, biochemistry, and physiology. The use of knockout mice lacking key signaling and regulatory proteins may also shed light on the molecular mechanisms underlying adaptation at photoreceptor synapses.

## Data availability statement

The raw data supporting the conclusions of this article will be made available by the authors, without undue reservation.

## Ethics statement

The animal study was approved by OHSU Institutional Animal Care and Use Committee. The study was conducted in accordance with the local legislation and institutional requirements.

## Author contributions

Study design: CM, TH. Experimentation: TH, CM, GR, SA, JC. Data analysis: CM, RH, RD. Figure and manuscript preparation: CM, RH, JC. Editing and review: CM, RH, JC, RD. All authors contributed to the article and approved the submitted version.
